# Human Milk Electrolytes as Nutritional Biomarkers of Mammary Gland Integrity: A Study Across Ductal Conditions and Donor Milk

**DOI:** 10.3390/nu17203283

**Published:** 2025-10-19

**Authors:** Po-Yu Hsieh, Miori Tanaka, Tomoko Himi, Katsumi Mizuno

**Affiliations:** 1 The Nippon Foundation Human Milk Bank, Tokyo 103-0016, Japan; miori.tanaka@milkbank.or.jp (M.T.); katsuorobi@med.showa-u.ac.jp (K.M.); 2 Department of Pediatrics, Showa Medical University School of Medicine, Tokyo 142-8555, Japan; 3 Division of Neonatology, Department of Pediatrics, Chang Gung Memorial Hospital, Taoyuan 33305, Taiwan; 4 Oketani Breastfeeding Management Research Institute, Tokyo 162-0044, Japan; himiko4716@gmail.com; 5 Japanese Human Milk Bank Association, Tokyo 103-8480, Japan

**Keywords:** sodium, electrolyte, human milk, donor milk, mammary gland integrity, lactational dysfunction, ductal obstruction

## Abstract

**Background/Objectives**: Sodium (Na) concentration and the sodium-to-potassium (Na/K) ratio in human milk reflect epithelial tight junction integrity and have been proposed as non-invasive biomarkers of lactational dysfunction, including subclinical mastitis and ductal obstruction. However, their discriminative performance across varied mammary duct conditions, as well as their relevance to milk quality and nutritional integrity, remain underexplored. This study aimed to evaluate the ability of Na, K and the Na/K ratio to discriminate ductal obstruction from non-obstructed lactation—including normal, mixed, and donor milk—and to assess their applicability as nutritional and clinical screening biomarkers. **Methods**: The study analyzed 635 human milk samples from four groups: obstructed ducts (*n* = 94), mixed ducts (*n* = 39), normal ducts (*n* = 102), and donor milk (*n* = 400). Na and K concentrations were measured using validated handheld ion-selective electrode analyzers. Statistical analyses included Quade’s ANCOVA and receiver operating characteristic curve analysis, adjusting for infant age, gestational age, birth body weight, maternal age and storage duration. **Results**: Na concentrations were highest in obstructed ducts (Group A: median 810 ppm, IQR 368–1725) compared with normal ducts (Group C: 220 ppm, IQR 140–283) and donor milk (Group D: 98 ppm, IQR 80–130) (*p* < 0.001). A similar pattern was observed for the Na/K ratio (Group A: 1.5, IQR 0.6–3.1 vs. Group C: 0.3, IQR 0.2–0.5; Group D: 0.3, IQR 0.2–0.3). After adjusting, both Na and the Na/K ratio remained significantly elevated in milk from obstructed ducts compared to non-obstructed samples (*p* < 0.001). Donor milk exhibited the lowest and most stable electrolyte levels. Na demonstrated excellent discriminative performance (area under the curve = 0.96), slightly outperforming the Na/K ratio (area under the curve = 0.92). **Conclusions**: Na concentration and the Na/K ratio in human milk are sensitive and practical biomarkers of mammary gland integrity. Given that Na alone can be measured without additional calculations, its simplicity and strong performance support its application as a potential biomarker for ductal obstruction, with implications for both lactation support and nutritional science.

## 1. Introduction

Human milk provides optimal nutrition for infants and plays a crucial role in neonatal growth, immunity, and development. In addition to macronutrients and immunological components, the electrolyte composition of human milk has gained increasing attention as a physiological indicator of lactation health and milk quality [1]. Among these electrolytes, sodium (Na) and the sodium-to-potassium (Na/K) ratio are particularly sensitive to the integrity of mammary tight junctions and are known to fluctuate in response to inflammation, secretory activation, or lactational dysfunction [2,3,4,5]. These biomarkers reflect changes in epithelial permeability and are considered useful for the assessment of lactation function and breast health [6,7,8]. As electrolyte shifts influence milk osmolality and the Na load delivered to infants, these biomarkers are directly relevant to nutritional physiology.

Mastitis, one of the most common complications during lactation, is often caused by milk stasis, inflammation, or infection and may occur as early as 2–3 weeks postpartum. Clinical symptoms include breast tenderness, erythema, swelling, fever, and systemic discomfort [9,10]. However, assessment remains largely clinical and subjective, as no standardized laboratory tests are routinely used. This can pose challenges, particularly for subclinical mastitis or for healthcare providers with limited lactation expertise. Inflammatory processes such as ductal obstruction or subclinical mastitis can alter tight junction permeability, leading to increased Na concentrations and Na/K ratios in milk. Prior studies of ionic composition have demonstrated that these ionic shifts may precede or accompany overt symptoms of mastitis. Elevated Na levels or Na/K ratios have been proposed as non-invasive, quantitative indicators of early breast inflammation [5,11,12,13]. Furthermore, emerging technologies such as portable ion-selective electrode analyzers now allow for accurate, point-of-care measurement of milk Na and K levels, offering a practical tool for real-time lactation assessment [14,15,16].

Despite growing interest in the role of milk electrolytes in lactation assessment, few studies have systematically compared these biomarkers across varying mammary duct conditions, such as obstructed, mixed, and normal ducts [17]. Moreover, although donor milk is generally considered physiologically mature and derived from established lactation [1], it has seldom been included as a reference group in such comparisons. These gaps limit the generalizability of previous findings, which have largely focused on early postpartum or pathological states and leave open questions about the discriminative performance of electrolyte biomarkers under broader lactational conditions. This study aimed to evaluate the utility of Na, K and Na/K ratio as nutritional biomarkers of ductal obstruction in human milk samples classified by localized duct condition. Using region-specific sampling guided by clinical assessment, the study further examined their discriminative performance in practical, real-world lactation care settings and to inform nutrition-oriented lactation support.

## 2. Materials and Methods

### 2.1. Study Design and Participants

This observational, cross-sectional study analyzed human milk samples collected between June and December 2024, including samples from mothers and donor milk from human milk banks. No restrictions were placed on infant gestational age (GA), so both term and preterm infants were naturally represented across the study groups. Samples were categorized into four groups: Group A (obstructed ducts) comprised milk samples expressed from nipple orifices affected by localized ductal obstruction, such as mastitis, blebs, or clogged ducts. These samples were classified based on visual and tactile examination. In cases of mastitis, the milk typically appeared yellow, viscous, and cloudy, and mothers frequently reported pain or tenderness during expression. Obstructed ducts are often presented with slightly turbid, watery milk resembling rice water, along with resistance or prickling pain during expression. Group B (mixed ducts) included milk that was unintentionally expressed from both obstructed and normal ducts due to anatomical proximity, resulting in a mixture. This occurred when localized expressions from obstructed ducts alone were not feasible. Group C (normal ducts) comprises milk from unaffected nipple orifices outside the affected duct. Finally, Group D consisted of donor milk obtained from the only two certified human milk banks in Japan, serving as a mature lactation reference. All donor mothers were exclusively breastfeeding, asymptomatic, and at least one month postpartum, with completed health screening and sufficient surplus milk (>1 L) to ensure stable and sufficient lactation. The donor selection criteria were based on the national guidelines for donor milk established by Ministry of Health, Labour and Welfare, Japan. Samples not meeting these criteria were not collected or provided to the study.

In Japan, there are currently two certified human milk banks: The Japan Human Milk Bank Association, founded in 2017, and The Nippon Foundation Human Milk Bank, established in 2021 in cooperation with the Association. Together, they coordinate milk banking nationwide, providing appropriately pasteurized donor milk to very low birth weight and other high-risk infants, while also supporting research on nutritional and bioactive components of human milk to inform neonatal care guidelines. The classification of Groups A to C was performed by nationally certified midwives affiliated with the Oketani Lactation Society in Japan. In Japan, certified midwives play an integral role in postnatal lactation care and are licensed to perform clinical breast assessments. Three midwives participated in sample classification and collection, including one internationally board-certified lactation consultants. Each sample evaluated by a pair of midwives with one performing breast massage and the other collecting milk. Diagnostic consistency was ensured through standardized criteria and prior training in visual and tactile assessment. The group assignment was performed at the sample level rather than the individual level, allowing for regional differentiation within a single participant. All available samples during the study period were included. For Groups A–C, no explicit exclusion criteria were applied apart from inability to provide milk at the time of assessment.

All participants and donors provided written informed consent and voluntarily contributed their milk samples for research purposes. The Institutional Review Board of Showa Medical University approved the study (IRB No. 2023-217-A).

### 2.2. Sample Collection and Handling

For Groups A to C, samples were collected manually by certified midwives using standardized breast massage techniques and measured on-site immediately after expression without storage or homogenization involved. Donor milk in Group D was obtained from donors following standardized collection and handling protocols, including hand expression using sterile containers. In addition, some donors used breast pumps (manual or electric, including single and double pumps) for milk expression; however, detailed pump type information was not consistently recorded and was therefore not included in the statistical analyses. After expression, donor milk was frozen and shipped under temperature-controlled conditions and stored at the milk bank under −30 °C. Prior to analysis, donor milk samples were thawed in a cold-water bath (10–20 °C) and homogenized using a high-intensity ultrasonic liquid processor (Miris Ultrasonic Processor; Sonics & Material Inc., Newtown, CT, USA) to ensure even distribution of fat and solutes. Donor milk samples were not pooled and originated from single donors.

### 2.3. Electrolyte Analysis and Variables

Na and K concentrations were measured using handheld direct ion-selective electrode analyzers (LAQUAtwin Na-11 and K-11; HORIBA Advanced Techno, Co., Ltd., Kyoto, Japan). The Na/K ratio was calculated for each sample. Concentrations were reported in parts per million (ppm), the default output unit of the analyzers. For reference or comparison with plasma electrolytes, values can be converted to mmol/L using the following conversion factors: 1 ppm Na ≈ 0.0435 mmol/L; 1 ppm K ≈ 0.0256 mmol/L All measurements were performed by trained personnel following the manufacturer’s instructions. Maternal and infant characteristics were recorded, including maternal age, GA at birth, birth weight (BW), and infant age at the time of milk expression. For Groups A–C, maternal age, GA, and BW were initially reported by the mothers through a questionnaire and subsequently verified against medical records by certified midwives, with the data entered into a standardized Microsoft Excel sheet. For Group D, donor mothers provided the information on registration forms, which was reviewed and confirmed by a physician during interview, and then organized into the database by milk bank coordinators. The data between milk expression and analysis was recorded for all donor milk samples to estimate storage duration.

### 2.4. Statistical Analysis

No a priori sample size calculation was conducted, as this was an observational study based on all available milk samples collected during the study period. Descriptive statistics were used to summarize maternal and infant characteristics, as well as concentrations of Na and K in milk across the four groups. Normality of continuous variables was assessed using the Shapiro–Wilk test. Continuous variables are presented as median and interquartile range (IQR) due to non-normal distribution. Group differences were analyzed using Kruskal–Wallis tests, followed by Bonferroni-adjusted pairwise Mann–Whitney U tests for pos hoc comparisons.

To examine the overall effects on Na, K, and the Na/K ratio across the four groups without specifying a reference group while adjusting for covariates (maternal age, GA, BW, infant age at milk expression, and storage duration), Quade’s ANCOVA was performed. This rank-based, nonparametric method was applied for non-normally distributed data while adjusting for multiple covariates. First, dependent variables were rank-transformed, and covariates were adjusted using multivariate linear regression, followed by univariate general linear modeling of the residuals to assess group effects. Bonferroni-corrected post hoc comparisons were applied. To clarify pairwise group differences, additional multivariate regression models using dummy-coded group variables were performed, with Group C (normal ducts) used as the reference category.

To further explore discriminative performance of electrolyte biomarkers in detecting ductal obstruction, subgroup analyses were conducted. First, Group A (obstructed ducts) was compared to a combined reference group of Group C and D (non-obstructed ducts). Group B (mixed ducts) was excluded due to heterogeneous mammary duct characteristics. As a sensitivity analysis, an additional regression was conducted comparing Group A to all remaining samples (Groups B, C, and D) to evaluate whether including mixed ducts affected discriminative performance. All models adjusted for the same maternal and infant covariates.

Receiver operating characteristics (ROC) curve analysis was performed to evaluate the discriminative ability of each biomarker. The area under the curve (AUC), optimal cutoff value, sensitivity, specificity and Youden index were calculated. Correlation analyses between milk electrolytes (Na, K, and the Na/K ratio) and clinical variables (maternal age, GA, BW, infant age, and storage duration) were assessed using Spearman’s correlation. Storage duration was only included in correlation analysis within Group D, as samples from Groups A–C were all freshly expressed.

Clinical data was extracted from medical records and manually entered into Microsoft Excel. All statistical analyses were performed using SPSS version 25 (IBM Corp., Armonk, NY, USA). Statistical significance was set at *p* < 0.05.

## 3. Results

### 3.1. Characteristics of Study Groups

A total of 635 human milk samples were analyzed and categorized into four groups: obstructed ducts (Group A, *n* = 94), mixed ducts (Group B, *n* = 39), normal ducts (Group C, *n* = 102), and donor milk from the human milk bank (Group D, *n* = 400).

Table 1 summarizes the maternal and infant characteristics, along with the concentrations of Na, K, and the Na/K ratio across the four groups. Significant group differences were observed in maternal age, GA at birth, infant age at milk expression (all *p* < 0.001) and infant BW (*p* = 0.001). Donors in Group D had the youngest median age (33 years; IQR 29–38) with broader donor population compared with Groups A–C (median 35–36 years; IQR 33–38). All study groups included both term and preterm infants, while Group D included mothers of very preterm infants, reflected by a broader GA range (IQR 29–39 weeks). Infant age at milk expression differed notably, with Group D expressing milk at a median of 5 months (IQR 3–7), compared with 2–3 months in Groups A–C. Storage duration was 0 days for all fresh samples (Groups A–C), while donor milk in Group D had a median storage of 124 days (IQR 82–150).

### 3.2. Na, K, and the Na/K Ratio Concentrations Across Groups

**Figure 1:**
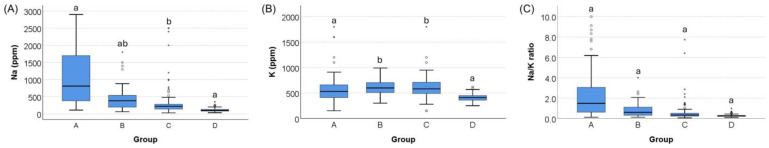
Distribution of Na (**A**), K (**B**), and the Na/K ratio (**C**) in human milk samples. Boxplots showed the median and interquartile range. Extreme values for Na and the Na/K ratio in Group A beyond the Y-axis limits have been truncated for clarity, while all data points were included in the statistical analyses. * and ◦ represent extreme and outlier values as identified by SPSS boxplot analysis. Different lowercase letters (a, b) indicate statistically significant differences between groups (*p* < 0.05) based on post hoc tests after adjusting for covariates using Quade’s ANCOVA.

Significant intergroup differences in Na, K, and the Na/K ratio were observed (all *p* < 0.001). Group A had the highest median Na concentration (810 ppm), followed by Group B (380 ppm). Groups C (220 ppm) and D (98 ppm) had the lowest values. Median Na levels in Group A were approximately 8-fold higher than in Group D (810 vs. 98 ppm). The Na/K ratio followed a similar pattern, with Group A showing the highest median (1.5), Group B intermediate (0.6), and Groups C and D lowest (0.3 for C and D). Notably, Group A demonstrated greater variability in Na and the Na/K ratio, reflected by wider IQRs (Na: 368–1725 ppm; Na/K ratio: 0.6–3.1) and more outliers, whereas Group D had the narrowest distribution (Na: 80–130 ppm; Na/K ratio: 0.2–0.3). In contrast, K concentrations were relatively consistent across groups, with slightly lower levels in Group D (median 410 ppm) compared to Groups A–C (median 530–600 ppm). These distributions are illustrated in Table 1 and Figure 1.

### 3.3. Group Comparison After Adjusting for Covariates

Using Quade’s ANCOVA, which adjusted for maternal age, GA, infant BW, infant age at milk expression, and storage duration, significant group effects were found for Na (F = 6.049, *p* < 0.001) and K (F = 19.429, *p* < 0.001). This shows that the observed differences across groups in raw comparisons (Table 1 and Figure 1) remained significant even after accounting for potential confounding factors. In contrast, the Na/K ratio did not differ significantly between groups after adjustment (F = 2.157, *p* = 0.092) (Table 2).

Table 3 presents the pairwise results of multivariate linear regression using Quade’s rank-transformed outcomes to further clarified the independent effects of group membership and maternal and infant factors on milk electrolytes. Compared with Group C (normal ducts), Na levels were significantly higher in Group A (B = 145.56, *p* < 0.001), Group B (B = 78.34, *p* = 0.001) and significantly lower in Group D (B = −123.39, *p* < 0.001). K concentrations were significantly lower in both Group A (B = −72.24, *p* = 0.001) and Group D (B = −232.37, *p* < 0.001). Regarding the Na/K ratio, Group A (B = 189.45, *p* < 0.001) and B (B = 95.61, *p* < 0.001) differed significantly from Group C,. Among covariates, higher maternal age was associated with slightly higher Na (B = 2.67, *p* = 0.024), while infant age at milk expression was significantly and negatively associated with rank-transformed Na (B = −3.96, *p* < 0.001) and K (B = −3.94, *p* = 0.007). Storage duration—relevant only to donor milk—was significantly and negatively associated with Na (B = −0.40, *p* < 0.001) and the Na/K ratio (B = −0.71, *p* < 0.001), but not with K (*p* = 0.095). Other covariates, including GA and BW, had minimal or nonsignificant effects across outcomes.

To further investigate factors contributing to electrolyte variation in donor milk (Group D), separate multiple linear regression analyses were conducted using infant age at milk expression and storage duration as independent variables. For Na, both infant age at milk expression (standardized β = −0.178, *p* < 0.001) and storage duration (β = −0.207, *p* < 0.001) were independently associated with lower concentrations. For K, only infant age showed a modest negative association (β = −0.131, *p* = 0.009), while storage duration had no significant effect (*p* = 0.266).

### 3.4. Discrimination Between Ductal Obstruction and Non-Obstructed Groups

Na and the Na/K ratio were significantly higher in Group A compared with the combined normal and donor milk group (Groups C and D), with adjusted coefficients of B = 196.86 and B = 192.37, respectively (both *p* < 0.001). K levels showed no significant difference (*p* = 0.243). Among covariates, infant age and storage duration were significantly and negatively associated with Na and K concentrations, while only storage duration showed a significant effect on the Na/K ratio. Maternal age was positively associated with Na and K levels. In contrast, GA and BW had limited or inconsistent effects across outcomes (Table A1).

**Figure 2:**
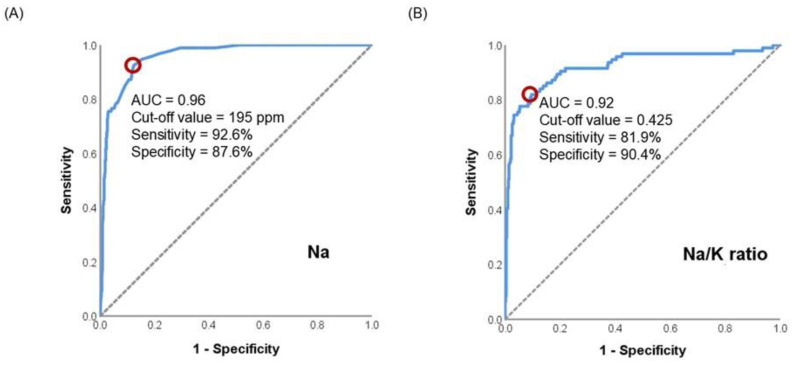
Receiver operating characteristic curves and optimal cutoff values of (**A**) Na and (**B**) the Na/K ratio in identifying ductal obstruction (Group A) from the combined normal and donor milk group (Groups C and D). The red circle indicates the optimal cutoff point determined by Youden’s index.

In primary ROC curve analysis shown in Figure 2, Na showed excellent discrimination between obstructed and non-obstructed ducts (Groups C and D) with an AUC of 0.96 (95% CI: 0.94–0.98, *p* < 0.001). The optimal cutoff of 195 ppm yielded 92.6% sensitivity and 87.6% specificity. The Na/K ratio also demonstrated strong discriminatory power (AUC: 0.92, 95% CI: 0.88–0.96, *p* < 0.001) with an optimal cutoff of 0.425 (sensitivity: 81.9%, specificity: 90.4%). In contrast, K had a limited performance (AUC: 0.67) and was thus excluded from the main figure.

In a supplementary model comparing Group A to all non-obstructed samples (Groups B + C + D), Na and the Na/K ratio remained significantly elevated (*p* < 0.001), mirroring prior results. Covariate effects were consistent with earlier findings (Table A2). Na maintained a strong discriminatory performance with an AUC of 0.942. The optimal cutoff of 195 ppm yielded a sensitivity of 92.6% and specificity of 83.2%. The Na/K also demonstrated good discriminatory value with an AUC of 0.908. Depending on the selected threshold, a cutoff of 0.57 achieved a sensitivity of 77.7% and specificity of 91.3%, while a stricter cutoff of 0.80 yielded slightly lower sensitivity (74.5%) but higher specificity (94.5%).

### 3.5. Correlations with Maternal and Infant Characteristics

**Figure 3:**
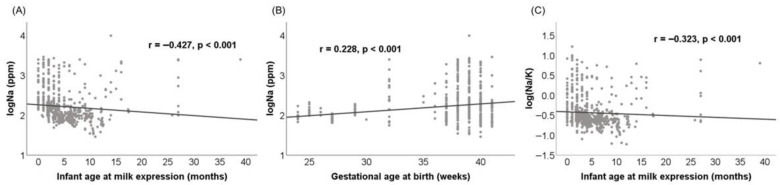
Correlations between (**A**) logNa and infant age at milk expression, (**B**) logNa and gestational age at birth, and (**C**) log(Na/K) and infant age at milk expression. Spearman’s correlation coefficients (r) and *p*-values are indicated on each panel. Na and the Na/K ratio values were log-transformed to improve point distribution.

Across all samples, maternal and infant characteristics exhibited modest but significant correlations with milk electrolytes. Na concentrations were moderately negatively correlated with infant age at milk expression (r = −0.427, *p* < 0.001) and weakly positively correlated with GA (r = 0.228), BW (r = 0.206), and maternal age (r = 0.172), which were all *p* < 0.001. The Na/K ratio showed similar results but with slightly weaker coefficients. In donor milk (Group D) specifically, storage duration was negatively correlated with both Na (r = −0.237) and the Na/K ratio (r = −0.309) (*p* < 0.001 for both), but not with K (*p* = 0.612), suggesting selective effects of freezing and storage on these analytes. K levels showed modest associations with several variables, including negative correlations with infant age, and positive correlations with GA and BW (Table A3). Figure 3 visualizes the strongest correlations for Na and the Na/K ratio using log-transformed values to reduce skewness. GA was included for its physiological relevance.

## 4. Discussion

In this study, we found that Na concentration and the Na/K ratio reflect mammary gland integrity across different ductal conditions and donor milk. Notably, Na alone showed excellent discriminative performance in distinguishing obstructed ducts from normal duct or donor milk, with an optimal cutoff of 195 ppm and slightly superior sensitivity compared with the Na/K ratio. These findings suggest that milk electrolytes can serve not only as clinical indicators of ductal health but also as nutritional biomarkers relevant to infant feeding. The observed associations persisted after adjustment for maternal and infant characteristics, highlighting the potential robustness of these markers in diverse lactational settings.

Human milk from obstructed ducts exhibited the highest levels of Na and the Na/K ratio, consistent with prior reports linking elevated Na to nutritionally relevant marker of lactational physiology and inflammatory changes in the mammary gland [7,18]. In contrast, donor milk, obtained from mothers with established, asymptomatic lactation, exhibited the lowest Na and Na/K ratio values with minimal variability, consistent with mature lactational physiology and tight junction closure [8,14]. These physiological patterns support prior findings that reduced Na and Na/K reflect intact mammary epithelium and effective milk secretion [19,20]. The intermediate values seen in mixed ducts further suggest a spectrum of glandular function, aligning with the concept of partial ductal compromise. This category has not been clearly defined in prior literature but may reflect a transitional or subclinical state in lactation physiology.

Our adjusted analyses confirmed that mammary duct group differences persisted for Na and K concentrations, but not for the Na/K ratio. This suggests that Na and K may better reflect underlying glandular changes beyond those attributable to infant BW or age at milk expression, which may have implications for nutritional quality and milk suitability in early feeding. These findings are consistent with earlier studies showing that milk Na is a reliable biomarker of epithelial integrity and inflammation [8,21], while the Na/K ratio may be more prone to variability due to infant-related factors or lactational stage [22]. This reinforces the potential utility of Na as a primary discriminative indicator in diverse clinical settings.

Regarding maternal and infant variables, infant age at expression was consistently and negatively associated with Na and K, reflecting the transition from early lactation to a more mature secretory profile, as tight junction closure occurs over time [1,23]. Maternal age also showed modest independent association with Na, while GA and BW showed relatively modest or inconsistent effects. This supports that electrolyte profiles in milk are more closely influenced by current glandular activity and lactational stage than by perinatal maturity such as GA or BW [22,24]. Notably, storage duration—applicable only to donor milk—was significantly associated with lower Na and the Na/K levels, though this likely reflects the inherent characteristics of mature donor milk and its processing, which is further discussed below.

In subgroup analyses, Na and the Na/K ratio consistently demonstrated effective biomarkers for distinguishing ductal obstruction, regardless of whether mixed ducts (Group B) were included in the reference group. This consistency reinforces their robustness and generalizability, suggesting that their diagnostic utilities are not overly sensitive to variations in comparison group definition.

ROC findings highlight the superior discriminative utility of Na compared with the Na/K ratio. Given that Na alone can be measured without additional calculations, it may offer a simple and practical option for identifying ductal obstruction or mastitis, with potential relevance for lactation management and milk bank screening. This aligns with earlier findings highlighting its utility as a quantitative diagnostic tool [14,21,25].

The observed negative correlation of Na and the Na/K ratio with infant age at expression suggests a maturation-related decline in these values as lactation progresses, consistent with physiological adaption to infant nutritional needs [14,24]. Maternal age, GA, and BW all showed weak positive correlations with Na and the Na/K ratio, reinforcing that current lactational stage and glandular activity may play a larger role in shaping electrolyte composition than perinatal factors [4,24].

Importantly, in contrast to previous studies relying on whole-breast sampling or postpartum timing, our study uniquely utilized region-specific classification of milk samples based on local mammary duct status assessed as obstructed, mixed, or normal by certified midwives. Despite this targeted method, our results demonstrated that Na retained strong discriminatory capacity even when obstructed samples were compared against all other non-obstructed ducts, including physiologically normal and donor milk. These findings suggest that milk Na levels may act as a nutritional biomarker even when assessed at localized ductal levels and remain discriminatively useful even without precise ductal localization, offering a nuanced understanding of intramammary variation.

Donor milk is often presumed to represent “normal” or mature lactation [1,8]. In our results indeed showed significantly lower value and variability of Na and K concentrations compared to milk expressed during early postpartum or from mothers with lactation-related issues, support its role as a stable physiological reference. The donor selection criteria, including the requirement for exclusive breastfeeding, and a minimum milk volume threshold, further strengthen this interpretation. By including certified donor human milk as a comparator, our study establishes physiologically mature milk profiles, enabling robust comparisons across diverse lactational states.

However, while donor milk is generally considered mature and homogeneous, our subgroup analysis limited to donor milk (Group D) revealed that both infant age at expression and storage duration may still subtly influence Na and the Na/K ratio levels. In particular, prolonged frozen storage may modestly reduce Na content, although these changes are relatively small and unlikely to impact clinical use. Our observation aligns with previous reports suggesting that while Na is relatively stable during storage, slight reductions in the Na/K ratio may occur with prolonged or repeated freezing conditions [5,26]. This discrepancy may reflect differences in the biological sources and stability of these electrolytes. Na levels largely depend on the integrity of mammary epithelial tight junctions, which can be altered by freezing and thawing processes, whereas K remains more stable within intracellular compartments. This finding highlights the importance of considering lactational stage and milk handling even in standardized donor milk, particularly when using these electrolytes as nutritional biomarkers, as well as interpreting donor milk data in studies comparing samples across different lactational stages.

### Strengths and Limitations

This study included a large cohort of 635 milk samples, allowing for the powerful evaluation of Na concentration and the Na/K ratio as nutritional biomarkers reflecting mammary gland integrity across different ductal conditions and donor milk. By demonstrating their value beyond clinical diagnostics, these findings expand the role of these electrolytes into the field of nutritional science. After adjusting for maternal and infant factors, Na and the Na/K ratio remained significantly elevated in samples from regions with ductal obstruction, supporting their potential as indicators of localized ductal inflammation. By incorporating four distinct groups, our study provides one of the most comprehensive assessments of human milk electrolyte composition and highlights the translational utility of Na analysis as a non-invasive biomarker of milk quality and integrity relevant to infant nutrition. In addition, electrolyte concentrations were measured using validated portable ion-selective electrode analyzers, providing a reproducible and practical approach for both research and nutritional monitoring.

This study also has some limitations. First, the classification of mammary duct status was based on clinical judgment by certified midwives, which may involve some degree of subjectivity despite the use of standardized criteria and shared training. Second, the cross-sectional design without serial sampling precludes assessment of dynamic changes in milk electrolyte composition over time. A longitudinal design would allow for the tracking of Na and the Na/K ratio across the course of mastitis development and resolution, potentially revealing temporal patterns of inflammation and recovery, and helping to identify optimal timing for intervention or nutritional monitoring. Third, although we adjusted for key maternal and infant variables, unmeasured factors such as maternal parity, sampling time, maternal hydration, breast feeding behavior (including milk collection methods such as manual expression versus mechanical pumping) or nutritional status could have influenced the results. Lastly, while donor milk served as a reference for mature lactation, its handling and storage procedures differed from freshly expressed milk, which may affect direct comparability. These limitations should be considered when interpreting electrolyte profiles as nutritional biomarkers of milk quality.

## 5. Conclusions

This study evaluated Na, K, and the Na/K ratio as nutritional biomarkers of ductal obstruction in human milk. Among the three biomarkers, Na concentration and the Na/K ratio showed excellent discriminative performance, while K provided limited utility. After adjusting for maternal and infant characteristics, both Na and the Na/K ratio remained significantly elevated in obstructed ducts, supporting their role as independent biomarkers of mammary gland integrity. These findings suggest that Na measurement alone may serve as a potential biomarker for duct obstruction. Future longitudinal studies are warranted to validate predictive value prior to clinical symptom onset.

## Figures and Tables

**Table 1:** Baseline characteristics of human milk samples by study group.

Variable	Group A (Obstructed) *n* = 94	Group B (Mixed) *n* = 39	Group C (Normal) *n* = 102	Group D (Donor Milk) *n* = 400	***p***
Maternal age, years	35 (33–38) ^a^	35 (33–38) ^ab^	36 (34–38) ^a^	33 (29–38) ^b^	**<0.001**
GA at birth, weeks	39 (38–39) ^a^	39 (38–39 )^a^	39 (37–40) ^a^	38 (29–39) ^b^	**<0.001**
Infant BW, g	2911 (2763–3258) ^a^	2966 (2708–3274) ^ab^	2968 (2763–3245) ^a^	2874 (904–3204) ^b^	**0.001**
Infant age, months	3 (1–5) ^a^	2 (1–4) ^a^	2 (1–3) ^b^	5 (3–7) ^c^	**<0.001**
Storage duration, days	0 (fresh sample) ^a^	0 (fresh sample) ^a^	0 (fresh sample) ^a^	124 (82–150) ^b^	**<0.001**
Na, ppm	810 (368–1725) ^a^	380 (190–550) ^b^	220 (140–283) ^c^	98 (80–130) ^d^	**<0.001**
K, ppm	530 (410–663) ^a^	600 (500–710) ^a^	580 (488–710) ^a^	410 (360–450) ^b^	**<0.001**
Na/K ratio	1.5 (0.6–3.1) ^a^	0.6 (0.3–1.1) ^b^	0.3 (0.2–0.5) ^c^	0.3 (0.2–0.3) ^d^	**<0.001**

Values are presented as median (interquartile range, IQR). Missing data for gestational age in 5 samples was excluded from the analysis. Superscript letters denote significant group differences (Bonferroni-adjusted Mann–Whitney U tests, *p* < 0.05). Values in bold indicate statistical significance (*p* < 0.05). GA—gestational age; BW—birth weight; Na—sodium; K—potassium; Na/K ratio—sodium-to-potassium ratio.

**Table 2:** Adjusted group differences in human milk sodium, potassium, and the Na/K ratio based on Quade’s ANCOVA.

Dependent Variable	Group Effect (F)	*p*
Sodium (Na)	6.049	**<0.001**
Potassium (K)	19.429	**<0.001**
Na/K ratio	2.157	0.092

Values in bold indicate statistical significance (*p* < 0.05).

**Table 3:** Multivariate linear regression using rank-transformed outcomes (Quade’s ANCOVA) assessing factors associated with human milk sodium, potassium, and the Na/K ratio.

Dependent Variable	Independent Variable	B (SE)	95% CI (Lower to Upper)	*p*
Rank Sodium (Na)	Group A vs. C	145.56 (17.87)	110.47 to 180.65	**<0.001**
	Group B vs. C	78.34 (23.25)	32.68 to 124.01	**0.001**
	Group D vs. C	−123.39 (19.24)	−161.17 to −85.61	**<0.001**
	Maternal age	2.67 (1.18)	0.36 to 4.98	**0.024**
	Gestational age at birth	−1.99 (3.13)	−8.13 to 4.15	0.524
	Infant birth weight	0.03 (0.02)	−0.006 to 0.06	0.119
	Infant age at milk expression	−3.96 (1.19)	−6.29 to −1.63	**<0.001**
	Storage duration	−0.40 (0.11)	−0.61 to −0.18	**<0.001**
Rank Potassium (K)	Group A vs. C	−72.24 (22.05)	−115.53 to −28.95	**0.001**
	Group B vs. C	4.01 (28.69)	−52.33 to 60.34	0.889
	Group D vs. C	−232.37 (23.73)	−278.98 to −185.77	**<0.001**
	Maternal age	1.83 (1.45)	−1.02 to 4.68	0.208
	Gestational age at birth	6.60 (3.86)	−0.98 to 14.18	0.088
	Infant birth weight	−0.01 (0.02)	−0.05 to 0.03	0.753
	Infant age at milk expression	−3.94 (1.47)	−6.82 to −1.07	**0.007**
	Storage duration	0.23 (0.14)	−0.04 to 0.49	0.095
Rank Na/K ratio	Group A vs. C	189.45 (20.96)	148.28 to 230.61	**<0.001**
	Group B vs. C	95.61 (27.28)	42.05 to 149.17	**<0.001**
	Group D vs. C	−7.27 (22.57)	−51.59 to 37.04	0.747
	Maternal age	2.12 (1.38)	−0.59 to 4.83	0.125
	Gestational age at birth	−6.09 (3.67)	−13.30 to 1.11	0.097
	Infant birth weight	0.03 (0.02)	0.002 to 0.07	0.066
	Infant age at milk expression	−2.24 (1.39)	−4.98 to 0.50	0.109
	Storage duration	−0.71 (0.13)	−0.97 to −0.46	**<0.001**

Group C (normal duct) is used as reference. Values in bold indicate statistical significance (*p* < 0.05). SE—standard error; CI—confidence interval.

## Data Availability

The datasets generated and analyzed during the current study are not publicly available due to restrictions imposed by the informed consent process and ethics approval. De-identified data may be available from the corresponding author upon reasonable request and subject to institutional approval.

## Abbreviations

The following abbreviations are used in this manuscript:

Na	Sodium
K	Potassium
Na/K	Sodium-to-Potassium
GA	Gestational Age
BW	Birth Weight
IQR	Interquartile Range
ROC	Receiver Operating Characteristics
AUC	Area Under the Curve

## Appendix A

**Table A1:** Multivariate linear regression using rank-transformed sodium, potassium, and Na/K ratio to compare ductal obstruction (Group A) with normal and donor milk samples (Groups C + D), adjusted for maternal and infant characteristics.

Dependent Variable	Independent Variable	B (SE)	95% CI (Lower to Upper)	*p*
Rank Sodium (Na)	Group A vs. C + D	196.86 (16.51)	164.43 to 229.29	**<0.001**
	Maternal age	4.95 (1.20)	2.59 to 7.31	**<0.001**
	Gestational age at birth	−1.18 (3.33)	−7.73 to 5.37	0.724
	Infant birth weight	0.02 (0.02)	−0.01 to 0.06	0.201
	Infant age at milk expression	−4.98 (1.33)	−7.59 to −2.37	**<0.001**
	Storage duration	−0.83 (0.09)	−1.00 to −0.65	**<0.001**
Rank Potassium (K)	Group A vs. C + D	24. 67 (21.10)	−16.77 to 66.11	0.243
	Maternal age	5.20 (1.53)	2.18 to 8.22	**0.001**
	Gestational age at birth	10.69 (4.26)	2.32 to 19.05	**0.012**
	Infant birth weight	−0.03 (0.02)	−0.069 to 0.016	0.225
	Infant age at milk expression	−5.74 (1.70)	−9.08 to −2.41	**0.001**
	Storage duration	−0.59 (0.11)	−0.82 to −0.37	**<0.001**
Rank Na/K ratio	Group A vs. C + D	192.37 (18.43)	156.17 to 228.57	**<0.001**
	Maternal age	2.66 (1.34)	0.03 to 5.30	**0.048**
	Gestational age at birth	−7.20 (3.72)	−14.51 to 0.11	0.053
	Infant birth weight	0.04 (0.02)	0.003 to 0.08	**0.033**
	Infant age at milk expression	−2.48 (1.48)	−5.39 to 0.44	0.095
	Storage duration	−0.73 (0.10)	−0.93 to −0.54	**<0.001**

Values in bold indicate statistical significance (*p* < 0.05). SE—standard error; CI—confidence interval.

**Table A2:** Multivariate linear regression comparing rank-transformed sodium, potassium, and Na/K ratio between ductal obstruction (Group A) with all non-obstructed samples (Groups B + C + D), adjusted for maternal and infant characteristics.

Dependent Variable	Independent Variable	B (SE)	95% CI (Lower to Upper)	*p*
Rank Sodium (Na)	Group A vs. B + C + D	172.89 (16.34)	140.80 to 204.99	**<0.001**
	Maternal age	5.12 (1.20)	2.77 to 7.47	**<0.001**
	Gestational age at birth	−0.25 (3.30)	−6.74 to 6.24	0.940
	Infant birth weight	0.02 (0.02)	−0.02 to 0.05	0.354
	Infant age at milk expression	−4.35 (1.25)	−6.81 to −1.89	**0.001**
	Storage duration	−0.99 (0.09)	−1.16 to −0.82	**<0.001**
Rank Potassium (K)	Group A vs. B + C + D	5.87 (20.64)	−34.66 to 46.40	0.776
	Maternal age	5.91 (1.51)	2.93 to 8.88	**<0.001**
	Gestational age at birth	9.15 (4.17)	0.96 to 17.34	**0.029**
	Infant birth weight	−0.02 (0.02)	−0.06 to 0.02	0.365
	Infant age at milk expression	−4.86 (1.58)	−7.97 to −1.75	**0.002**
	Storage duration	−0.72 (0.11)	−0.93 to −0.51	**<0.001**
Rank Na/K ratio	Group A vs. B + C + D	174.17 (18.30)	138.23 to 210.12	**<0.001**
	Maternal age	2.60 (1.34)	−0.03 to 5.24	0.053
	Gestational age at birth	−5.52 (3.70)	−12.79 to 1.74	0.136
	Infant birth weight	0.03 (0.02)	0.006 to 0.07	0.099
	Infant age at milk expression	−2.15 (1.40)	−4.90 to 0.61	0.127
	Storage duration	−0.86 (0.10)	−1.05 to −0.67	**<0.001**

Values in bold indicate statistical significance (*p* < 0.05). SE—standard error; CI—confidence interval.

**Table A3:** Spearman’s correlation between milk electrolytes and variables.

Variable	Na (r)	Na (*p*-Value)	K (r)	K (*p*-Value)	Na/K (r)	Na/K (*p*-Value)
Maternal age	0.172	**<0.001**	0.147	**<0.001**	0.109	**0.006**
Gestational age at birth	0.228	**<0.001**	0.318	**<0.001**	0.110	**0.006**
Infant birth weight	0.206	**<0.001**	0.228	**<0.001**	0.133	**0.001**
Infant age at milk expression	−0.427	**<0.001**	−0.300	**<0.001**	−0.323	**<0.001**
Storage duration *	−0.237	**<0.001**	−0.025	0.612	−0.309	**<0.001**

* Only applicable to donor milk (Group D, *n* = 400). Values in bold indicate statistical significance (*p* < 0.05). Na—sodium; K—potassium.
